# Diabetes Distress in Young Adults with Type 2 Diabetes: A Cross-Sectional Survey in China

**DOI:** 10.1155/2020/4814378

**Published:** 2020-06-18

**Authors:** Yanfen Hu, Lingxia Li, Jun Zhang

**Affiliations:** ^1^School of Health, Wuhan University, Wuhan, Hubei 430071, China; ^2^The Cadre Ward, The Second Affiliated Hospital of Xi'an Jiaotong University, Xi'an, Shaanxi 710004, China

## Abstract

**Background:**

Diabetes distress is a negative emotion related to diabetes management, which can compromise self-care and management of diabetes. However, few studies on diabetes distress have focused on young adults with type 2 diabetes in China.

**Methods:**

A cross-sectional survey was conducted. Using a convenient sampling method, 98 young adults with type 2 diabetes who were admitted to our hospital from June 2017 to July 2018 were selected as research subjects. They were investigated using a basic demographic questionnaire, Diabetes Distress Scale, Summary of Diabetes Self-Care Activities Measure, and Audit of Disease Knowledge. Pearson's correlation analysis and regression analysis were used to analyze the influencing factors of diabetic distress.

**Results:**

Among participants, 90.82% suffered from diabetes distress with an average score of 3.01 ± 0.58. Regimen-related, emotional burden-related, and interpersonal-related distress were the most frequently reported as severe. The results of the single-factor analysis showed that gender (*P* = 0.019), age (*P* = 0.003), occupation (*P* = 0.022), smoking (*P* < 0.001), and diabetes complications (*P* = 0.001) were the main factors affecting diabetes distress. The correlation analysis showed that diabetes distress was negatively correlated with the level of diabetic self-management (*P* < 0.001, *r* = −0.377) but not with the level of diabetes knowledge (*P* = 0.052, *r* = −0.197). The results of a multiple regression analysis showed that self-management level (*P* = 0.001, 95% CI: -0.039-0.011), age (*P* = 0.002, 95% CI: -0.463-0.104), smoking (*P* = 0.018, 95% CI: -0.504-0.048), and complications (*P* = 0.009, 95% CI: -0.517-0.076) accounted for 35.42% of the total variation in diabetes distress.

**Conclusion:**

Young adults with type 2 diabetes reported severe diabetes distress. Age, smoking, and diabetes complications were the main factors influencing diabetes distress in young adults with type 2 diabetes. Results of the present study are fundamental in selecting targeted measures for alleviating diabetes distress and thus improving the quality of life in these patients.

## 1. Introduction

Diabetes distress is a negative emotion related to diabetes management. This distress can compromise self-care and management of diabetes [1]. A meta-analysis study (*n* = 36, 998) found that the overall prevalence of diabetes distress is 36% in people with type 2 diabetes [2]. In Guangzhou, China, the prevalence of diabetes distress among those with type 2 diabetes is as high as 77.23% [3]. This distress does not resolve over time and often remains chronic without intervention [4]. Few studies on diabetes distress have looked at Chinese young adults with type 2 diabetes. As young people are of great importance to society, it is crucial to investigate diabetes distress in these young adults to identify the influencing factors. Doing so can help promote interventions to alleviate diabetes distress and improve quality of life among young people.

According to the 18th edition of the International Diabetes Federation's global overview of diabetes released in 2017, nearly 500 million people have diabetes, including 121 million in China [5]. Among them, type 2 diabetes accounts for more than 90% of diagnoses, and the incidence among young people is increasing. Studies have shown that 42.5%–77.23% of diabetic patients suffer from diabetes distress [3, 6–8]. This distress affects mental health, blood glucose monitoring, and self-management [9], and it is negatively correlated with health-related quality of life [10, 11]. In a study from China, diabetes distress had indirect effects on glycemic control through diabetes self-efficacy and self-management [12]. Diabetes self-efficacy also indirectly affected glycemic control through diabetes self-management [12]. Diabetes distress thus might help predict depression and could be an important factor in treatment adherence [7]. To investigate the diabetes distress in young adults with type 2 diabetes and analyze its influencing factors is fundamental in selecting targeted measures for alleviating diabetes distress and thus improving the quality of life in these patients.

## 2. Materials and Methods

### 2.1. Study Design

A cross-sectional survey was carried out adhering to the Strengthening the Reporting of Observational studies in Epidemiology (STROBE) guidelines for observational studies.

### 2.2. Setting, Participants, and Variables

Using a convenient sampling method, we selected 98 young adults with type 2 diabetes who were admitted to the hospital from June 2017 to August 2018. Inclusion criteria were as follows: (1) informed and voluntary participation in the study, (2) between 18 and 55 years old, and (3) meets the diagnostic criteria of the Chinese guidelines for the prevention and treatment of type 2 diabetes (2013 edition). Exclusion criteria were as follows: (1) history of mental illness or communication disorders; 2) serious liver, kidney, or other organ dysfunction; 3) undergoing radiotherapy, chemotherapy, or organ transplantation; and (4) serious complications of diabetes, such as dialysis to maintain life. This study was approved by the Ethics Committee of our hospital, and was conducted in accordance with the Declaration of Helsinki. All patients and their families gave informed consent, participated voluntarily, and signed informed consent forms.

### 2.3. Data Measurement

Baseline data were collected upon admission. Sociodemographic characteristics and medical conditions were collected using self-designed questionnaires. Diabetes distress was assessed using the Diabetes Distress Scale (DDS) [13]. The DDS, developed by Polonsky et al. [13], has four subscales: emotional burden (5 items), physician-related distress (4 items), regimen-related distress (5 items), and diabetes-related interpersonal distress (3 items). Each item is scored from 1 to 6. A mean item score < 2 indicates no distress, 2–2.9 indicates moderate distress, and >3 indicates severe distress. The scale was translated into Chinese by Qing and Xueqin [14]. Cronbach's alpha coefficient was 0.842–0.951 [14].

To assess self-management, we used the Summary of Diabetes Self-care Activities [15]. This survey has 11 items, including diet (questions 1–4), exercise (questions 5–6), blood glucose monitoring (questions 7–8), foot care (questions 9–10), and smoking status (question 11). Each item is scored on a scale from 0 to 7. The higher the total score, the higher the level of diabetes self-management. Question 4 is scored backwards. Li and Weiping [16] translated the scale to Chinese and verified its reliability and validity. Overall Cronbach's alpha coefficient of the scale is 0.918 [16].

To assess knowledge of diabetes, we used the Audit of Diabetes Knowledge, [17] which was imported and localized by Weiyan from Zhejiang University [18]. It contains 26 items in eight subscales related to diabetes topics: treatment; illness; hypoglycemia; physical exercise; complications; influences of smoking, alcohol, and food; foot care; and diet. Each item has three answer options: “right,” “wrong,” and “do not know.” One point is awarded for a correct answer and zero points for a wrong answer or not knowing. The scale has good reliability and validity, with Cronbach's alpha coefficient of 0.909 and content validity of 0.923.

### 2.4. Statistical Methods

Data were analyzed using SPSS 19.0 software. The results are expressed as the mean ± standard error of the mean $\left(\bar{x}\pm S\right)$. Two independent sample *t*-tests were used to compare between two groups, and ANOVA and LSD tests were used to compare three or more groups. A Pearson correlation analysis was used to analyze the correlation between diabetes distress, self-management level, and diabetes knowledge. The influencing factors of diabetic pain were analyzed using multiple regression. *P* < 0.05 was considered statistically significant.

## 3. Results

### 3.1. Diabetes Distress among Young Adults with Type 2 Diabetes

Among participants, 90.82% (89) patients were suffering diabetes distress. Of those, 57.14% (56) had severe diabetes distress, 33.67% (33 cases) had moderate diabetes distress, and 9.18% (9) had no diabetes distress. The average score of diabetes distress among the 98 patients was 3.01 ± 0.58. Three subscales showed severe levels of distress: regimen-related, emotional burden, and diabetes-related interpersonal distress. Physician-related distress was reported as moderate (see Table 1 for details).

### 3.2. Univariate Analysis of Factors Affecting Diabetes Distress

Table 2 shows the sociodemographic characteristics and medical conditions of the 98 participants with type 2 diabetes. The univariate analysis showed that gender, age, occupation, smoking status, and complications were the main factors affecting diabetes distress (*P* < 0.05). Most smokers were male, so we analyzed the effect of smoking on diabetes distress in male patients only. The results showed that diabetes distress in smoking patients was significantly higher than that in nonsmoking patients (Table 3, *P* < 0.05).

### 3.3. Correlation between Self-Management, Knowledge, and Diabetes Distress

To determine the relationship between self-management, knowledge of diabetes, and diabetes distress, we performed a Pearson correlational analysis. We found a negative and significant correlation between self-management level and the diabetes distress score (*r* = −0.377, *P* < 0.001, Table 4). We found no significant correlation between knowledge of diabetes and diabetes distress (*r* = −0.197, *P* = 0.052, Table 4).

### 3.4. Factors Contributing to Diabetes Distress

We conducted multiple regression analyses on self-management, gender, age, occupation, smoking, and complications to determine factors contributing to diabetes distress. The results showed that self-management level, age, smoking, and complications diabetes were determinants of the diabetes distress scores, accounting for 35.42% of total variance in diabetes distress scores (Table 5, *R*^2^ = 0.3542).

## 4. Discussion

Young adults with diabetes are prone to diabetes distress, perhaps because they also must manage family, social, and work responsibilities. Diabetes distress in young patients is reportedly higher than it is among elderly patients [19]. In our study population of young adults with type 2 diabetes, 90.82% suffered from diabetes distress, with an average score of 3.01 ± 0.58. Among them, 57.14% had severe diabetes distress, and 33.67% had moderate diabetes distress. We also found that diabetes distress among young adults with type 2 diabetes has psychological characteristics related to family and social roles. For example, young people often have many responsibilities, such as supporting their children and elderly family members, handling financial- and career-related responsibilities, and managing their households. These stressors can increase the burdens associated with diabetes. Alleviating psychological distress in young adults with type 2 diabetes thus is extremely important to improve their quality of life.

Regimen-related distress and emotional burden are two important measures of distress in patients with type 2 diabetes [19, 20]. In addition to rating these two measures as severe, participants in our study also scored diabetes-related interpersonal distress as severe. This additional measure may be due to interpersonal communication needs related to their careers. For example, young people often engage in work-related social activities to advance their careers, but the dietary restrictions, lifestyle habits, and other behaviors associated with diabetes management can affect participation in these activities. Some of these lifestyle behaviors are hard to maintain and thus often ignored. Patients with type 2 diabetes also may feel that they do not get enough emotional support from family and friends. This lack of support can lead to low self-esteem, depression, and social phobia.

Previous studies have shown that many factors influence diabetes distress, such as sleep, exercise, diet, and treatment regimens [6]. In the present study, we found that gender, age, occupation, smoking, and complications were the main factors affecting diabetes distress in young adults with type 2 diabetes. Education level, duration of diabetes, and family income had no significant influence on diabetes distress, which differs from findings in previous reports.

In this study, men reported significantly higher diabetes distress than women, which differs from previous reports [2]. This difference might be due to unique family and social responsibilities and stress among men in modern society. We also found that younger patients had significantly higher levels of diabetes distress than older patients, which is in accordance with previous findings [21]. In China, most patients over the age of 45 have less pressure to provide for their children's education, mortgage, and car loan. Their work also tends to be stable, and they have fewer family burdens and societal pressures. In addition, there were many patients with diabetes in the same age group, and they were more likely to receive understanding and emotional support from family and friends. Peer support has been proven to be effective in reducing diabetes distress in patients with type 2 diabetes mellitus [22].

Previous reports indicate that education and family income are important factors influencing psychological distress among diabetic patients [23]. However, we found that education and family income had no significant influence on diabetes distress. In the past, it was believed that patients with higher education levels also had higher diabetes knowledge. However, with the popularity of smart phones and rapid development of social media, young and middle-aged patients can easily obtain diabetes-related knowledge, which greatly reduces the association between diabetes knowledge and education. With the popularization of the new rural cooperative medical care system and residents' medical insurance in China, the cost of diabetes treatment is no longer a huge burden for patients. This may be an important reason why education and family income are no longer important influences on the psychological distress of diabetic patients.

Few studies have examined the effects of smoking on diabetes distress. We found that the pain level reported by male, diabetic smokers was significantly higher than that reported by male, diabetic nonsmokers. Further studies are needed to identify the causes of this increased pain. We speculate that it may be related to the level of self-management among these patients.

Previous studies have shown significant differences in diabetes distress reported by patients with different occupations. For example, diabetes distress in farmers is higher than that reported in patients with other occupations [24]. We found that those working in enterprise/institution fields had the highest levels of diabetes distress (3.20 ± 0.09), whereas farmers reported the lowest levels (2.74 ± 0.14). Perhaps nowadays farmers tend to have less occupational stress and less housing pressure than enterprise/enterprise employees.

In this study, the correlation analysis showed that diabetes distress was negatively correlated with self-management, which is consistent with previous reports [25, 26]. However, no correlation between diabetes distress and knowledge of diabetes was observed, indicating that all young adults in the study had some knowledge of diabetes, but the transformation from “knowing” to “doing” needs to be strengthened according to the knowledge-attitude-belief-practice model. The regression analysis showed that self-management, age, smoking, and complications of diabetes accounted for 35.42% of total variance in the diabetes distress scores. Treatment of this patient population thus should focus on achieving behavioral changes and promoting self-management, thus promoting mental health.

The limitation of this study was the limited sample size. In addition, as society develops and incidence of type 2 diabetes increase, young people with this disease may exhibit new diabetes distress characteristics. Continuous studies should assess other larger population samples to identify the incidence and influencing factors for diabetes distress.

## 5. Conclusion

Gender, age, occupation, smoking, and complications were the main factors affecting diabetes distress in young adults with type 2 diabetes. Among them, self-management, age, smoking, and diabetes complications accounted for 35.42% of the total variation in diabetes distress. Results of the present study are fundamental in selecting targeted measures for alleviating diabetes distress and thus improving the quality of life in these patients.

## Figures and Tables

**Table 1:** Score of diabetes distress of the 98 patients with type 2 diabetes $\left(\bar{x}\pm S\right)$.

Subscale	Item average score
Regimen-related distress	3.10 ± 0.91
Emotional burden	3.19 ± 1.00
Diabetes-related interpersonal distress	3.18 ± 0.89
Physician-related distress	2.26 ± 0.80
Average score of diabetes distress	3.01 ± 0.58

**Table 2:** Sociodemographic characteristics and medical condition of 98 participants with type 2 diabetes.

Variables	*n*	%	Score of diabetes distress($\bar{\text{x}}$ ± S)	*t*/*F*	*P*
Gender				*t* = 2.393	0.019^∗^
Male	65	66.33	3.10 ± 0.07		
Female	33	33.67	2.81 ± 0.09		
Age				*F* = 6.132	0.003^∗^
18-30	10	10.20	3.02 ± 0.01		
31-45	56	57.14	3.11 ± 0.08		
46-55	32	32.65	2.74 ± 0.09		
Marital status				*t* = 0.486	0.628
No spouse	10	10.20	3.00 ± 0.06		
Spouse	88	89.80	3.09 ± 0.20		
Education				*F* = 0.983	0.378
Junior high school	23	23.47	3.15 ± 0.12		
Senior high school	38	38.78	2.95 ± 0.09		
College and above	37	37.76	2.97 ± 0.09		
Occupation				*F* = 3.012	0.022^∗^
Farmer	13	13.27	2.74 ± 0.14		
Employees of enterprises/institutions	44	44.90	3.20 ± 0.09		
Self-employed entrepreneurs	19	19.39	2.78 ± 0.12		
Public servants	4	4.08	2.87 ± 0.38		
Others	18	18.37	2.99 ± 0.10		
Income (month)				*F* = 0.985	0.403
RMB 5000 or below	16	16.33	3.00 ± 0.09		
RMB 5000-10000	31	31.63	3.12 ± 0.10		
RMB 10000-15000	40	40.82	2.90 ± 0.10		
RMB15000 or more	11	11.22	3.11 ± 0.18		
Medical payment				*t* = 0.091	0.927
Self-paying	4	4.08	3.03 ± 0.06		
Medical insurance	94	95.92	3.01 ± 0.07		
Smoking				*t* = 3.676	<0.001^∗^
Yes	53	54.08	3.19 ± 0.07		
No	45	45.92	2.79 ± 0.09		
Drinking				*t* = 0.307	0.76
Yes	51	52.04	3.02 ± 0.09		
No	47	47.96	2.99 ± 0.08		
Duration of diabetes (year)				*F* = 2.271	0.109
<5	46	46.94	3.09 ± 0.08		
5-10	39	39.80	3.01 ± 0.10		
>10	13	13.27	2.71 ± 0.10		
Complications				*t* = 3.389	0.001^∗^
Yes	64	65.31	3.16 ± 0.07		
No	34	34.69	2.78 ± 0.09		
Type of treatment				*F* = 0.139	0.87
Oral hypoglycemic agents	34	34.69	3.04 ± 0.10		
Insulin	24	24.49	3.02 ± 0.14		
Insulin and oral	40.00	40.82	2.97 ± 0.08		

**Table 3:** Effect of smoking on the diabetes distress in male patients with type 2 diabetes (*n* = 65).

Variables	*n*	%	Score of diabetes distress $\left(\bar{x}\pm S\right)$	*t*/*F*	*P*
Yes	47	47.96	3.23 ± 0.07	2.806	0.007^∗^
No	18	18.37	2.78 ± 0.16		

**Table 4:** Correlation between self-management level, knowledge level of diabetes, and diabetes distress score (*n* = 98).

Item	Self-management level		Knowledge level of diabetes	
	*r*	*P*	*r*	*P*
Diabetes distress score	-0.377	<0.001	-0.197	0.052

**Table 5:** Multiple regression analysis for diabetes distress (*n* = 98).

Variables	Coefficients	SE	*t*	*P*	95% CI	
					Lower	Upper
Constant	4.787	0.358	13.354	<0.001	4.075	5.499
Self-management level	-0.025	0.007	-3.445	0.001^∗^	-0.039	-0.011
Gender	-0.032	0.122	-0.261	0.795	-0.274	0.210
Age	-0.283	0.090	-3.131	0.002^∗^	-0.463	-0.104
Occupation	0.070	0.049	1.439	0.154	-0.027	0.166
Smoking	-0.276	0.115	-2.400	0.018^∗^	-0.504	-0.048
Complications	-0.297	0.111	-2.669	0.009^∗^	-0.517	-0.076

## Data Availability

The data used to support the findings of this study are available from the corresponding author upon request.
